# Agricultural Practices Influence *Salmonella* Contamination and Survival in Pre-harvest Tomato Production

**DOI:** 10.3389/fmicb.2018.02451

**Published:** 2018-10-16

**Authors:** Ganyu Gu, Laura K. Strawn, David O. Oryang, Jie Zheng, Elizabeth A. Reed, Andrea R. Ottesen, Rebecca L. Bell, Yuhuan Chen, Steven Duret, David T. Ingram, Mark S. Reiter, Rachel Pfuntner, Eric W. Brown, Steven L. Rideout

**Affiliations:** ^1^Eastern Shore Agricultural Research and Extension Center, Virginia Tech, Painter, VA, United States; ^2^Center for Food Safety and Applied Nutrition, U.S. Food and Drug Administration, College Park, MD, United States

**Keywords:** *Salmonella*, tomato fields, irrigation, poultry litter, agricultural practices

## Abstract

Between 2000 and 2010 the Eastern Shore of Virginia was implicated in four *Salmonella* outbreaks associated with tomato. Therefore, a multi-year study (2012–2015) was performed to investigate presumptive factors associated with the contamination of *Salmonella* within tomato fields at Virginia Tech’s Eastern Shore Agricultural Research and Extension Center. Factors including irrigation water sources (pond and well), type of soil amendment: fresh poultry litter (PL), PL ash, and a conventional fertilizer (triple superphosphate – TSP), and production practices: staked with plastic mulch (SP), staked without plastic mulch (SW), and non-staked without plastic mulch (NW), were evaluated by split-plot or complete-block design. All field experiments relied on naturally occurring *Salmonella* contamination, except one follow up experiment (worst-case scenario) which examined the potential for contamination in tomato fruits when *Salmonella* was applied through drip irrigation. Samples were collected from pond and well water; PL, PL ash, and TSP; and the rhizosphere, leaves, and fruits of tomato plants. *Salmonella* was quantified using a most probable number method and contamination ratios were calculated for each treatment. *Salmonella* serovar was determined by molecular serotyping. *Salmonella* populations varied significantly by year; however, similar trends were evident each year. Findings showed use of untreated pond water and raw PL amendment increased the likelihood of *Salmonella* detection in tomato plots. *Salmonella* Newport and Typhimurium were the most frequently detected serovars in pond water and PL amendment samples, respectively. Interestingly, while these factors increased the likelihood of *Salmonella* detection in tomato plots (rhizosphere and leaves), all tomato fruits sampled (*n* = 4800) from these plots were *Salmonella* negative. Contamination of tomato fruits was extremely low (< 1%) even when tomato plots were artificially inoculated with an attenuated *Salmonella* Newport strain (10^4^ CFU/mL). Furthermore, *Salmonella* was not detected in tomato plots irrigated using well water and amended with PL ash or TSP. Production practices also influenced the likelihood of *Salmonella* detection in tomato plots. *Salmonella* detection was higher in tomato leaf samples for NW plots, compared to SP and SW plots. This study provides evidence that attention to agricultural inputs and production practices may help reduce the likelihood of *Salmonella* contamination in tomato fields.

## Introduction

Fresh produce is recognized as a potential vehicle for the transmission of foodborne pathogens (Brandl, 2006; Franz and van Bruggen, 2008c; Painter et al., 2013). *Salmonella enterica* is the leading cause of produce bacterial foodborne outbreaks in the United States, causing an estimated 1.4 million cases of illness and 500 deaths each year with a total estimated cost of $3.4 billion/year (Centers for Disease Control and Prevention [CDC], 2006, 2008). Consumption of *Salmonella* contaminated produce has led to several domestic multistate and international salmonellosis outbreaks (Centers for Disease Control and Prevention [CDC], 2005, 2007; Greene et al., 2008; Bennett et al., 2015). In addition, a considerable number of food recalls have occurred in past years as a result of contamination with *Salmonella* (Food and Drug Administration [FDA], 2017). Moreover, several tomato-associated *Salmonella* outbreaks have occurred, possibly due to in-field contamination (Hanning et al., 2009; Micallef et al., 2012; Bennett et al., 2015; Bell et al., 2015). Understanding potential routes of pathogen contamination of tomatoes is important.

Virginia is annually ranked in the top 10 states for fresh market tomato production (United States Department of Agriculture [USDA], 2009; Statista, 2015). In each salmonellosis outbreak that has been linked to tomatoes grown in Virginia, a specific pulsed field gel electrophoresis (PFGE) pattern of *S. enterica* serovar Newport (*S.* Newport JJPX01.0061) was implicated (Greene et al., 2008; Bennett et al., 2015). This PFGE pattern has also been isolated in *Salmonella* surveillances previously performed by the Food and Drug Administration (FDA) at Virginia Tech’s Eastern Shore Agricultural Research and Extension Center (ESAREC) (Bell et al., 2015). The repeated isolation of this strain from this region has raised concerns about its environmental fitness and persistence. Further studies are needed to determine the prevalence, persistence, and environmental sources of *S.* Newport JJPX01.0061.

Prior studies have highlighted the importance of water and soil amendments on pre-harvest contamination of produce (Brandl, 2006; Strawn et al., 2013; Bell et al., 2015). Contaminated irrigation water has the potential to play an important role in disseminating foodborne pathogens onto vegetables and fruits (Solomon et al., 2002; Islam et al., 2004a,b; Hintz et al., 2010). When irrigated with contaminated water, pathogens may adhere to plant surfaces, including fruits, flowers, and other edible parts. When present on produce, pathogens may persist and multiply at subsequent points along the farm-to-fork continuum (Solomon et al., 2002; Franz and van Bruggen, 2008c; Miles et al., 2009; Barak et al., 2011; Gu et al., 2011, 2013; Danyluk, 2013; Zheng et al., 2013). Additionally, multiple studies have demonstrated that foodborne pathogens can also be introduced in soil by the application of manure and then be transferred to plants in fields (Natvig et al., 2002; Solomon et al., 2002; Islam et al., 2004a; Ohtomo et al., 2004; You et al., 2006; Franz et al., 2008a,b; Semenov et al., 2009; Park et al., 2012). It has been reported that manure application can increase the likelihood of *Salmonella* contamination in soil (Strawn et al., 2013). Several studies worldwide have also indicated that fresh poultry litter (PL) could serve as a reservoir for various pathogenic organisms found in broiler farms, including *Salmonella* spp. (Opara et al., 1992; Pope and Cherry, 2000; van Asselt et al., 2009; Volkova et al., 2009; Suresh et al., 2011). Heat-treated or otherwise processed soil amendments may reduce the likelihood of introducing pathogens to the growing environment. For example, PL ash, which has higher phosphorous concentration than PL (Reiter and Middleton, 2016) and is used as a phosphorous (P_2_O_5_) source for agricultural crops in this region (Codling et al., 2002), can be sterilized during the burning process, where temperatures can exceed 266°C (Habetz and Echols, 2006; Clements, 2016).

Due to food safety concerns associated with infield release of pathogenic organisms, studies designed to illuminate the likelihood of contamination and transmission of *Salmonella* in pre-harvest production environments have seldom been conducted. In this study, irrigation water from a naturally contaminated pond at the ESAREC and fresh PL from naturally contaminated local broiler farms on the Eastern Shore of Virginia (ESV) were used as sources to investigate survival and transmission of *Salmonella* in tomato fields. Therefore, the goal of this study was to elucidate agricultural factors (water sources, type of amendments, and production practices) that influence *Salmonella* contamination in tomato fields at the ESAREC.

The objectives of the four experiments performed in this multi-year study were to: (1) quantify the likelihood and level of naturally occurring *Salmonella* contamination in tomato fields (including rhizosphere, leaf, and fruit samples) differing in irrigation water source (pond and well water) and production practice [staked with plastic mulch (SP), staked without plastic mulch (SW), and non-staked without plastic mulch (NW)]; (2) quantify the likelihood and level of naturally occurring *Salmonella* contamination in tomato fields amended with three independent soil amendments [fresh PL, PL ash, and conventional fertilizer triple superphosphate (TSP)], grown under the same three production practices in experiment 1 (SP, SW, and NW); (3) quantify the likelihood and level of naturally occurring *Salmonella* contamination in tomato fields amended with PL, PL ash, and TSP using two different irrigation water sources (pond and well water) under one production practice (SP); and (4) quantify the likelihood and level of contamination by an artificially inoculated (through drip irrigation) attenuated *Salmonella* Newport in tomato rhizosphere, on leaves, and on fruits under three different production practices (SP, SW, and NW).

## Materials and Methods

### Tomato Field Set Up and Experimental Design

The 4-year study was performed in experimental fields at the ESAREC, located in a major tomato and vegetable producing area of Virginia. The popular commercial tomato cultivar ‘BHN 602’ (BHNSeed, Immokalee, FL, United States) was used for this study. Tomato seeds were sowed in potting mix (Premier Horticulture Inc., Quebec, QC, Canada) and grown in a greenhouse located on-site (ESAREC). The temperature in the greenhouse ranged from 23 to 33°C, with an average temperature of 28°C during transplant production. Approximately 7-week-old tomato seedlings were transplanted to experimental fields at the ESAREC. Tomato plants were cared for according to Virginia Cooperative Extension recommendations (i.e., pest management and fertilization, etc.) (Wilson et al., 2012).

Four field experiments were performed during the 2012 to 2015 growing seasons (**Table 1** and **Supplementary Table S1**) to investigate the concentrations of recoverable *Salmonella* in tomato fields cultivated under different agricultural practices, which included use of two irrigation water sources (pond and well water), three fertilizers/soil amendments [PL, PL Ash, and a conventional fertilizer (Triple superphosphate, TSP)], and three production practices (SP, SW, and NW). Plastic mulch used in this study was a 0.03-mm-thick virtually impermeable polyethylene film (Berry Plastics Corp., Evansville, IN, United States). Irrigation was conducted by pumping water from the pond or well, both located on-site at the ESAREC. Plants were irrigated daily (∼ 6 L water/sub-plot) with either pond- or well-water through Aqua-Traxx drip tapes (TORO, Riverside, CA, United States) throughout the growing season, except on days with sufficient precipitation. Soil amendments/fertilizers were applied 1 week before transplanting by incorporation into soil to achieve recommended nutrition levels (Wilson et al., 2012). Twenty-five kilograms of fresh PL, 4 kg PL ash, or 2 kg TSP were applied to each corresponding sub-plot to provide a standard rate of 100 kg /hectare available P_2_O_5_ according to Virginia Cooperative Extension fertility recommendations for tomato (Maguire and Heckendorn, 2015). The average soil nitrogen concentrations (NO3-N) in experimental fields were 1.12, 0.59, and 4.84 mg/kg at 0–25, 25–50, and 50–75 cm depth, respectively. After fertilization and growing tomatoes in the field, soil nitrate rates would only be expected to change to 4.62, 2.66, and 2.22 mg/kg at 0–25, 25–50, and 50–75 cm depth, respectively, (unpublished data, available upon request).

**Table 1:** Set-up of the four tomato field experiments on *Salmonella* contamination.

Experiment	Contamination	Irrigation source	Type of soil amendment^a^	Production practices^b^	Number of repetition (year)
1	Natural contamination	Pond/ Well ^∗^	TSP	SP, SW, NW ^∗∗^	3 (2012, 2013, 2014)
2	Natural contamination	TSP	PL/PL ash/TSP ^∗^	SP, SW, NW ^∗∗^	4 (2012, 2013^c^, 2014, 2015)
3	Natural contamination	Pond/ Well ^∗^	PL/TSP ^∗∗^	SP	2 (2014, 2015)
4	Artificial inoculation of an attenuated *Salmonella* strain	Well	TSP	SP, SW, NW	1 (2014)

*^a^Fresh poultry litter (PL), PL ash, and conventional fertilizer (triple superphosphate – TSP); ^b^staked with plastic mulch (SP), staked without plastic mulch (SW), non-staked without plastic mulch (NW); ^c^Salmonella was not detected from tested samples in the 2013 field trial of experiment 2; ^∗^main split plot; ^∗∗^sub split plot.*

Experiment 1 was conducted annually during the growing seasons from 2012 to 2014 using a split-plot design with irrigation water source as the main plot factor (pond and well) and production practices as the sub-plot factor (SP, SW, and NW), with each combination replicated 4 times (total of 24 sub-plots, **Table 1** and **Supplementary Table S1**). Experiment 2 was arranged in a split-plot design with fertilization source as the main plot treatment (PL, PL Ash, and TSP) and production practices as the sub-plot factor. In 2012 and 2013 for experiment 2, two production practices (SP and NW) were used as sub-plot factors, with each fertilization/production practice combination replicated 4 times (total of 24 sub-plots). In 2014 and 2015 for experiment 2, three production practices (SP, SW, and NW) were examined, with each fertilization/production practice combination replicated 4 times (total of 36 sub-plots). Experiment 3 was conducted in 2014 and 2015 and arranged in a split-plot design with irrigation water source (pond or well) as the main plots and fertilizer source (PL or TSP) as the sub-plots, with each combination replicated 4 times (total of 16 sub-plots). Tomato plantings in experiment 3 were staked and covered with plastic mulch (SP), which is the typical production practice for tomato production on the ESV. In experiment 4, an attenuated, kanamycin-resistant *Salmonella* Newport strain SN#17 was obtained from the Microbiology division of the Center for Food Safety and Nutrition (CFSAN), FDA (Allard et al., 2014). The attenuated strain was incorporated into the tomato plots through injection into the existing irrigation drip tapes using a Chemilizer fixed ratio injector (CH9000-210, Hydro Systems, Cincinnati, OH, United States) in the middle of August one time at the blossoming stage. Approximately 2.5 L (10^4^ CFU/ml) bacterial solution was inoculated to each sub-plot (7.5 m^2^) through drip tape placed on the soil surface of tomato beds with emitters spaced every 30 cm along the tape. The total population of attenuated strain for each sub-plot approximated 2.5 × 10^7^ CFU. This field experiment was performed in a separated experimental plot in 2014 by following the biosafety standard operating protocols approved by the Institutional Biosafety Committee at Virginia Tech (Permit No.: IBC # 17-051). It was arranged in a randomized complete block design with four replications per different production practice (SP, SW, and NW) for a total of 12 *Salmonella*-inoculated sub-plots. The same number of plants grown in plots using three different production practices and all drip irrigated by non-inoculated well water were used as controls.

Each sub-plot was 12.5 m in length and 0.6 m in width and contained approximately 30 tomato plants. A 3 m border row between all sub-plots was set up to reduce the cross contamination between treatments.

A HOBO micro station (Onset Computer Corporation, Bourne, MA, United States) was set up to record the average temperature and total rainfall over the course of the 4-year study (monthly detection in 2012–2013, and weekly in 2014–2015).

### Irrigation Water Sampling

Pond and well water used for irrigation at the ESAREC were sampled at the beginning of each month to detect native *Salmonella* populations from August 2012 to December 2013. In 2014 and 2015, these water sources were sampled weekly. At each sampling time, 30 L (monthly detection) or 4 L (weekly detection) water samples were collected and stored on ice in the field prior to transport to the laboratory for analysis. Well water was pumped from ground, while pond water was collected from the surface area near the irrigation water intake.

*Salmonella* concentrations in water samples were determined using a previously described MPN method (Luo et al., 2014, 2016). In brief, three volumes of water samples (2000/500/100 mL for monthly samples, and 500/100/10 mL for weekly samples) were added to equal volumes of double strength lactose broth (LB, BD Biosciences, San Jose, CA, United States) with 12 and 4 replicates, respectively, and incubated at 37°C overnight. A 1 mL aliquot of each culture was subsequently transferred into 9 mL Tetrathionate Broth (TT Broth, Dot scientific inc., Burton, MI, United States) for selective enrichment at 37°C overnight. TT broth cultures were then streaked for isolation onto *Salmonella* selective XLT4 (BD Biosciences, San Jose, CA, United States) agar plates and incubated at 37°C overnight. Presumptive colonies (color black) were confirmed by the cross-streaking method using CHROMagar^TM^ *Salmonella* plates (DRG International Inc., Springfield, NJ, United States). *Salmonella* concentrations (MPN/liter) were determined by using MPN calculator build 23, created by Mike Curiale. The limits of quantitation were 0.03 to 25 MPN/liter for monthly sample analyses and 0.41 to 140 MPN/liter for weekly sample analyses, respectively, as determined by the range of the MPN analysis. Up to four confirmed colonies from each positive plate (as determined by the cross-streaking method) were stored in 20% glycerol at a −80°C freezer.

### Poultry Litter Sampling for Field Experiments With PL Application

Fresh PL and PL ash samples were analyzed for native *Salmonella* population via MPN analysis prior to usage in field experiments 2 and 3. In 2012, 2500 g composite samples of fresh PL were collected from each of the 14 houses of three broiler farms on the ESV (farms A, J, and D) for *Salmonella* detection before the field trial. In 2013, moist fresh PL collected from spaces under drinking-water pipes for flocks, and dry fresh PL collected from spaces between the drinking-water pipes were sampled from one broiler chicken farm on the ESV and processed for detection of *Salmonella* spp. PL ash composites were collected from four different factories located in Virginia and Pennsylvania and tested for *Salmonella* before field application. During 2014 and 2015, 500 kg of fresh PL (naturally contaminated with *Salmonella*) was collected from one broiler farm on ESV and used as soil amendment for fertilization in the field trials (Gu et al., 2015).

Similar to the water analyses, three volumes of PL samples (50, 10, and 1 g), each collected in octuplicate, were assessed for *Salmonella* population density as described above. The lower and upper detection limits of the MPN assay for the PL and PL ash were 2.1 to 2,100 MPN/kg, respectively. Up to four confirmed colonies from each positive plate were stored in 20% glycerol at a −80°C freezer.

### Tomato Rhizosphere Sampling and *Salmonella* Detection

Plant rhizosphere samples were collected monthly during the field experiments from 2012 to 2015 and processed for detection of *Salmonella* spp. At each sampling point, 10-g of soil was collected from 30 different areas of the same sub-plot using pre-sterilized soil probes (Oakfield Apparatus, Inc., Fond du Lac, WI, United States) and combined to form three composite samples (100 g/sample). A total of 12 samples (3 composite samples/sub-plot × 4 replications) were taken at each sampling time from each treatment in each trial of all four field experiments. 75% ethanol was used to disinfect soil probes between sampling.

Three portions of plant rhizosphere (50, 10, and 1 g) were included in the MPN scheme to assess the *Salmonella* population. The same plating and cross-streaking methods as mentioned above were used for *Salmonella* isolation and confirmation. Experiment 4 was an exception, which substituted trypticase soy agar (TSA) plates containing 50 μg/ml kanamycin for isolation of the attenuated *Salmonella* strain SN#17. The lower and upper detection limit for *Salmonella* was 1.4 to 2,500 MPN/kg, respectively, for the rhizosphere samples. Up to four confirmed colonies from each positive plate were stored in 20% glycerol in a −80°C freezer.

### Tomato Leaf Sampling and *Salmonella* Detection

Similar to rhizosphere sampling, tomato leaf samples were collected monthly during the field experiments from 2012 to 2015 for *Salmonella* detection. Composite sampling was used in each treatment per each trial. At each sampling time, 10 g of plant leaves were randomly collected with sterilized scissors from the 30 plants in each sub-plot to obtain three 100 g composite leaf samples. Leaves in each composite sample were chopped with sterilized scissors and stomached by Stomacher 400 Circulator (Seward Laboratory Systems Inc., Davie, FL, United States) in the sterile pre-labeled BA6141/CLR closure bags (Seward), and then transferred to equal volumes of double-strength LB. The same plating and cross-streaking methods as mentioned above were performed for *Salmonella* isolation and confirmation. Experiment 4 was the exception, where TSA plates containing 50 μg/ml kanamycin were used to isolate the attenuated *Salmonella* strain SN#17. Up to four confirmed colonies from each positive plate were stored in 20% glycerol in a −80°C freezer.

### Tomato Fruit Sampling and *Salmonella* Detection

To mimic the production of commercial tomato growers on ESV, tomato fruits were harvested twice from each field trial. At each sampling time, 25 mature red fruits were randomly collected from each sub-plot. Mature red fruits were removed and placed into a sanitized pre-labeled harvesting bin. One disinfected bin was used per sub-plot. A total of 200 fruits (25 fruits/sub-plot × 4 replications × 2 sampling times) were sampled for each treatment in each field trial for *Salmonella* detection. All the bins with harvested fruit were stored in a walk-in cold storage room at 12–15°C overnight prior to microbial analysis. For *Salmonella* detection, harvested fruits were placed in sterile pre-labeled BA6141/CLR closure bags (Seward) and stomached by Stomacher 400 Circulator in the bags to release the liquid exudate. In the 2012 field trials of experiments 1 and 2, and experiment 4 in 2014, each harvested fruit was tested individually for *Salmonella* presence. After the 1st year of individual fruit sampling (2012), to save labor and testing cost, fruits were pooled (3–5 fruits pooled in one sampling bag) for the remaining field trials of experiments 1–3 (2013–2015). Ten milliliters of fruit puree per bag was then added to equal volumes of double-strength LB and incubated at 37°C overnight. The same plating and cross-streaking methods as mentioned above were performed for *Salmonella* isolation and confirmation. TSA plates with 50 μg/ml kanamycin were used to recover attenuated *Salmonella* strain SN#17 in experiment 4. Up to four confirmed colonies from each positive plate were stored in 20% glycerol at a −80°C freezer. In total, 4,800 tomato fruits were harvested from plots applied with naturally contaminated sources (pond water and PL, experiments 1–3), and 600 fruits from inoculated plots (experiment 4) for *Salmonella* detections (**Table 1** and **Supplementary Table S1**).

### Molecular Serotyping Analysis

Up to two stored *Salmonella* isolates from each positive plate were selected for serotyping analysis using the CDC standard protocol for the molecular determination of *Salmonella* serotype (Centers for Disease Control and Prevention [CDC], 2009). Briefly, DNA from a pure culture was isolated using Instagene (BioRad, Hercules, CA, United States). Multiplex PCR was set up using Qiagen HotStar Master Mix (Qiagen) and 1 μL of DNA, and thermocycled under the following conditions: 95°C, 15 min.; 30 cycles of 94°C for 30 s, 48°C for 90 s, 72°C for 90 s; then 72°C for 10 min. DNA from the PCR reactions were then hybridized to the beads (xMAP^®^ *Salmonella* serotyping assay, Luminex) containing specific O- and H-Ag probes before the addition of strepavidin-R-phycoerythrin (Invitrogen div. Life Technologies, Grand Island, NY, United States). After incubation the samples were read using the Bio-Plex instrument (BioRad, Hercules, CA, United States). Positives were determined based on the ratio of signal to noise using a negative control (no template DNA) as a baseline. Serotype was determined based on which antigens are positive for each sample. *S. enterica* serovar Typhimurium strain ATCC 14028 was used as positive control.

### Statistical Analysis

With MPN analysis, a value of zero MPN was assigned to any samples under the detection limits. Upper limit values were given to any samples over the detection limits. Due to the low population densities of *Salmonella* in filed samples, MPN data were convert to MPN/kg or MPN/L for following analyses. The Mann–Whitney *U*-test, as a non-parametric test, was used to compare *Salmonella* concentrations in all experiments, except for when Student’s *t*-test was used to compare *Salmonella* MPN values and the contamination ratio in rhizosphere samples (as normal distribution) between pond water irrigated plots in experiment 1 and PL amended plots in experiment 2. Correlation of *Salmonella* MPN values between pond water and plant rhizosphere samples in experiment 1 was analyzed by Pearson correlation. The average temperature of the sampling month/week as well as the total rainfall 1 month/week prior to the monthly/weekly sampling point were calculated and compared with *Salmonella* prevalence data in irrigation pond water samples by correlation analysis. The interaction and effects of irrigation water source, fertilizer type, and/or production practice on the presence of *Salmonella* in tomato fields were analyzed by general mixed (GLIMMIX) model.

Statistical analysis was performed using SAS (SAS release 9.3, SAS Institute Inc., Cary, NC, United States). Except when stated otherwise, *P*-values of <0.05 were considered statistically significant.

## Results

### Prevalence and Diversity of *Salmonella* in Irrigation Water

**FIGURE 1:**
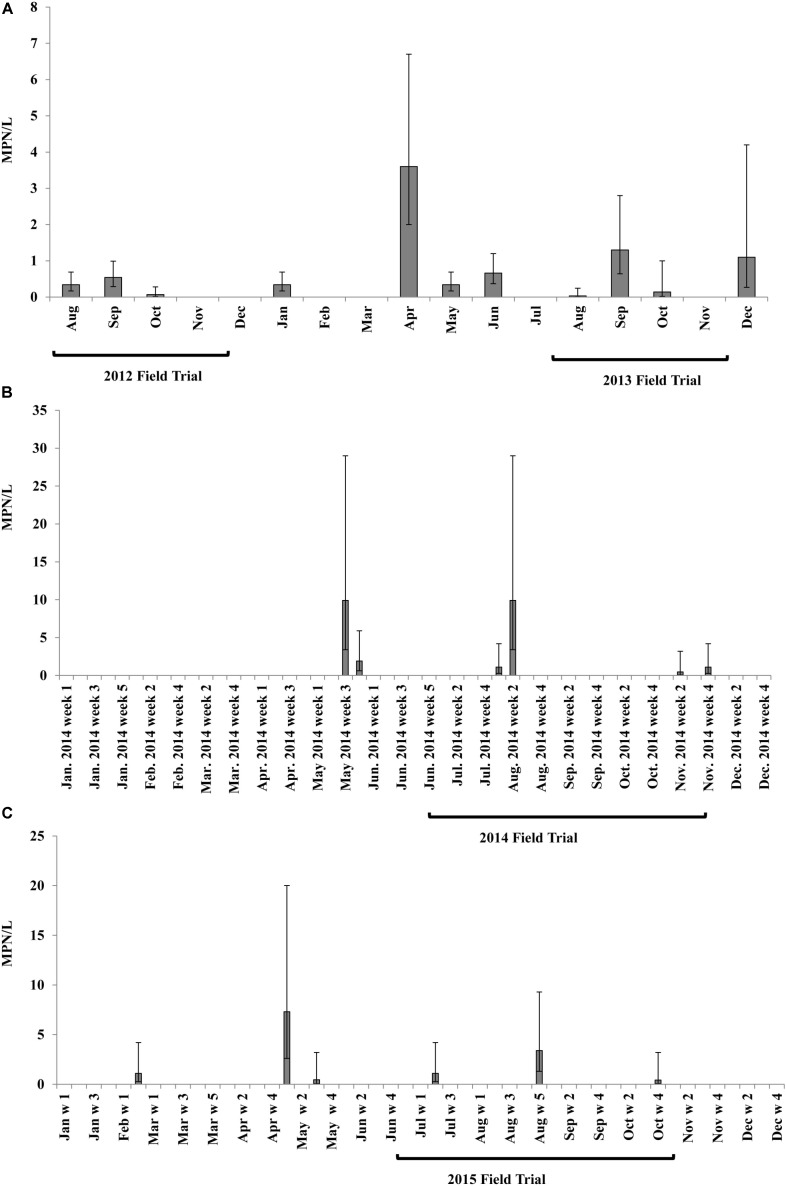
Most probable number (MPN) values of *Salmonella* spp. in irrigation pond water at Virginia Tech ESAREC from August 2012 to December 2013 **(A)** monthly detection; in 2014 **(B)** weekly detection; and in 2015 **(C)** weekly detection. Bars present 95% confidence intervals. Brackets cover the range of growth seasons of field trials performed in the 4-year study.

There were temporal (monthly or weekly) differences for *Salmonella* occurrence and concentration in irrigation pond water (**Figure 1**). The prevalence of *Salmonella* in pond water was 64.7% from August 2012 to December 2013 (11 of 17 months, **Figure 1A**), 11.8% in 2014 (6 of 51 weeks, **Figure 1B**), and 12.2% in 2015 (6 of 49 weeks, **Figure 1C**). The average *Salmonella* population density in positive pond water samples from August 2012 to December 2013 was 0.77 ± 0.31 MPN/L, and the average *Salmonella* concentrations in 2014 and 2015 were 4.06 ± 1.86 MPN/L and 2.30 ± 1.10 MPN/L, respectively.

*Salmonella* was isolated from the irrigation pond water during every growing season in the 4-year study. The highest observed *Salmonella* populations in pond water during irrigation occurred in September in 2012 (0.54 MPN/L, 95% confidence interval (CI): 0.29–0.99 MPN/L) and 2013 (1.3 MPN/L, 95% CI: 0.64–2.8 MPN/L) field trials, and in August 2014 (9.9 MPN/L, 95% CI: 3.4–29 MPN/L) and 2015 (3.4 MPN/L, 95% CI: 1.3–9.3 MPN/L) trials. In general, Newport was identified to be the most prevalent serovar of *Salmonella* isolated from pond water samples (**Supplementary Figure S1D**). However, the proportion of *Salmonella* Newport of identified isolates collected from pond water at the ESAREC decreased from 60 to 28% from 2013 to 2015 during the sampling periods examined in this study (**Supplementary Figures S1A–C**).

There were no significant correlations between weather parameters (temperature and rainfall) and *Salmonella* occurrence or MPN values in irrigation pond water during the study (*P* > 0.05).

No *Salmonella* was isolated from irrigation well water in this study.

### *Salmonella* Population Densities (MPN/kg) and Diversity in Poultry Litter Samples

**FIGURE 2:**
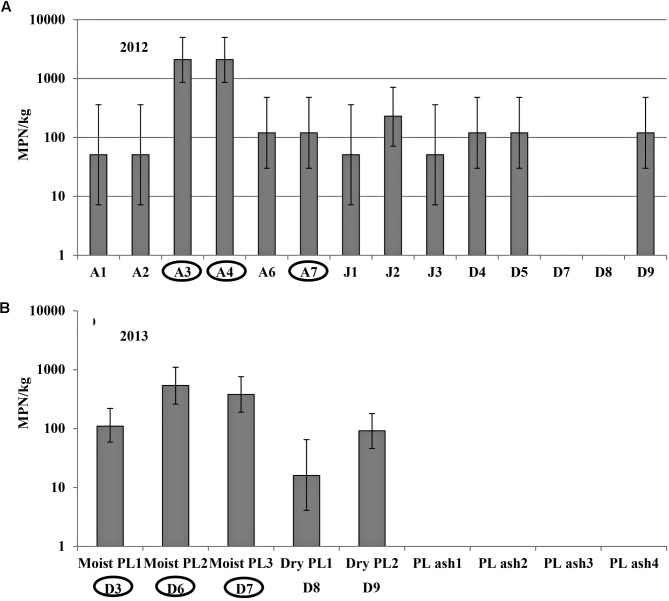
*Salmonella* population density (MPN/kg) in poultry litter (PL) samples in different broiler farms tested in 2012 **(A)** and in different PL and PL ash samples in 2013 **(B)**. Letters represent different chicken farms, and numbers behind represent different chicken houses in each farm. PL samples were collected from 14 chicken houses in 3 broiler farms in 2012 **(A)**. In 2013, moist and dry PL was sampled from 5 chicken houses in broiler farm D. PL ash was collected from 4 factories for *Salmonella* detection **(B)**. Fresh PL was collected from the circled chicken houses for soil amendment in 2012 **(A)** and 2013 **(B)** field trials of experiment 2. Bars represent 95% confidence intervals of *Salmonella* MPN values of tested samples.

*Salmonella* MPN levels in fresh PL samples collected from three broiler farms A, D, and J in 2012 varied in the 14 tested chicken houses, ranging from non-detectable (<2.1 MPN/kg) to maximum detection limit of 2100 MPN/kg (**Figure 2A**). Newport (*n* = 27 isolates), Saintpaul (*n* = 18), and Typhimurium (*n* = 8) were the top three *Salmonella* serovars isolated from fresh PL samples (*n* = 70) (**Supplementary Figure S2A**). Additional fresh litter from chicken houses A3, A4, and A7 was collected as fertilizer for soil amendment and applied to the field in 2012, prior to transplant.

In 2013, moist and dry fresh PL samples were collected from broiler chicken farm D, and PL ash from four different factories for *Salmonella* detection (**Figure 2B**). There were no significant differences in *Salmonella* MPN values between moist and dry PL samples (*P* > 0.05) gathered from the same farm, although it should be noted that the % moisture was not analyzed and all samples were reported on a wet-weight basis. The average population density of *Salmonella* in sampled fresh PL ranged from 16 to 540 MPN/kg (wet weight basis, **Figure 2B**). No *Salmonella* was isolated from PL ash samples (below detection limit of 2.1 MPN/kg). PL from chicken houses D3, D6, and D7 (circled in **Figure 2B**) were collected for use as the soil amendment in the field experiment in 2013. Typhimurium was the only *Salmonella* serovar identified from the fresh PL samples used for soil amendment in 2013 (*n* = 40).

Fresh PL was collected from broiler farm D on ESV in 2014 and 2015 as soil amendment for fertilization in the according field trials. *Salmonella* concentration in fresh PLs ranged from 34 MPN/kg (17–69 MPN/kg) in 2014 to 700 MPN/kg (270–1,800 MPN/kg) in 2015. Typhimurium (50%) and Kentucky (37%) were the two dominant *Salmonella* serovars isolated from PL in 2014 field trial (*n* = 39) (**Supplementary Figure S2B**), while serovars Typhimurium (45%), Newport (19%), Kentucky (16%), and Saintpaul (8%) were isolated from PL used for 2015 field trial (*n* = 49) (**Supplementary Figure S2C**).

No *Salmonella* was isolated from PL ash samples tested in this study.

### *Salmonella* Recovered From Tomato Fields Under Different Irrigation and Production Practices (Experiment 1)

**FIGURE 3:**
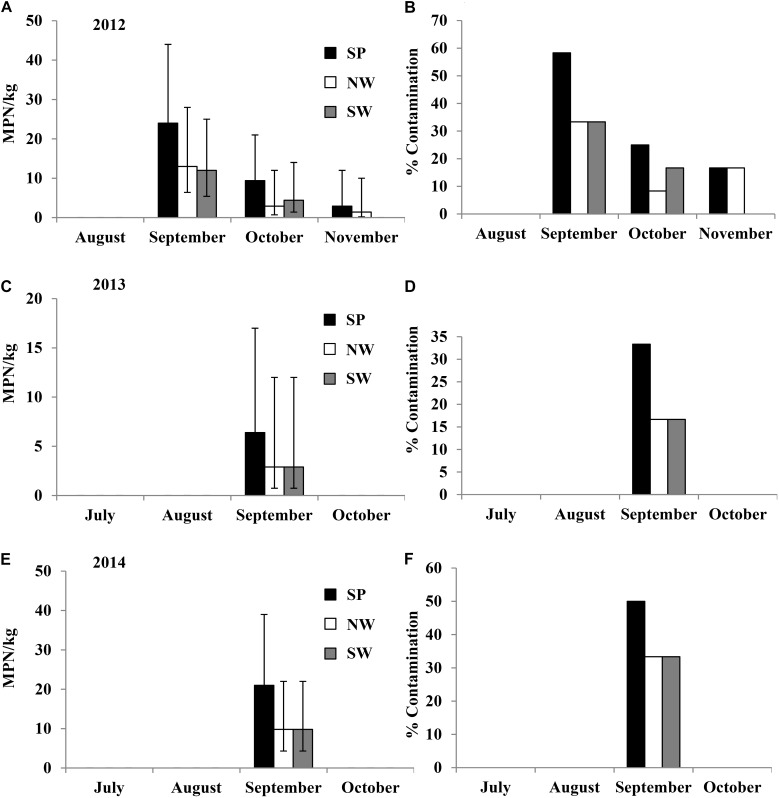
*Salmonella* population density (in 2012 **(A)**, 2013 **(C)**, and 2014 **(E)** field trials) and percent contaminated (in 2012 **(B)**, 2013 **(D)**, and 2014 **(F)** field trials) plant rhizosphere samples from pond water irrigated plots under different production practices in experiment 1: staked with plastic mulch (SP), staked without plastic mulch (SW), and non-staked without plastic mulch (NW). Bars represent the 95% confidence intervals of *Salmonella* MPN values in plant rhizosphere.

In the 2012 field trial for plots irrigated with pond water, *Salmonella* population density and contamination ratio in the plant rhizosphere increased from August to September and then subsequently reduced for the duration of the trial (**Figures 3A,B**). The highest average *Salmonella* population densities in the rhizosphere were 24 (95% CI: 13–44), 13 (95% CI: 6.4–28), and 12 (95% CI: 5.4–25) MPN/kg under production practices of SP, NW, and SW, respectively. *Salmonella* population density and contamination proportion per month in rhizosphere were relatively higher in the plots covered with plastic mulch (SP) compared with the other two plant practices (NW and SW). *Salmonella* was isolated from the leaves of tomato plants in NW plots only in September with an 8.3% contamination ratio (1/12, **Supplementary Table S2**). Newport was the only *Salmonella* serovar identified from plant rhizosphere and leaf samples collected in the 2012 field trial.

In the 2013 field trial, *Salmonella* was only isolated from plant rhizosphere in the plots that were irrigated with pond water, and only in September, with average population densities of 6.4 (95% CI: 2.4–17 MPN/kg), 2.9 (95% CI: 0.74–12 MPN/kg), and 2.9 (95% CI: 0.74–12 MPN/kg) MPN/kg under production practices of SP, NW, and SW, respectively (**Figures 3C,D**). No *Salmonella* was isolated from the leaf samples in 2013. *Salmonella* serovars Newport, Typhimurium, and Kentucky were isolated from the rhizosphere samples in tomato plots irrigated by pond water in 2013.

In 2014, similar to the 2013 trial, *Salmonella* was isolated from plant rhizosphere samples collected in September from plots irrigated by pond water. The average population densities were 21 (95% CI: 11–39 MPN/kg), 9.8 (95% CI: 4.3–22 MPN/kg), and 9.8 (95% CI: 4.3–22 MPN/kg) MPN/kg under production practices of SP, NW, and SW, respectively (**Figures 3E,F**). *Salmonella* was also isolated from the leaves of the tomato plants in NW plots in September with a contamination ratio of 33% (4/12 leaves examined, **Supplementary Table S2**). Only *Salmonella* Newport was isolated from the samples collected from plots irrigated by pond water in 2014.

In all three field trials of experiment 1, *Salmonella* was not isolated from the well water or the plants or plant rhizosphere in well irrigated plots. There were, however, significant positive correlations between *Salmonella* population densities in pond water and plant rhizosphere samples from pond water irrigated plots (**Table 2**).

**Table 2:** Correlation between *Salmonella* most probable number values (MPN) in plant rhizosphere (MPN/kg) and irrigation pond water (MPN/L) samples tested during the field trials of experiment 1.

MPN/L	Month	Pond water	Staked with mulch	Non-staked without mulch	Staked without mulch
2012 trial	August	0.34	0	0	0
	September	0.54	24	13	12
	October	0.068	9.4	2.9	4.4
	November	0	2.9	1.4	0
2013 trial	July	0	0	0	0
	August	0.034	0	0	0
	September	1.3	6.4	2.9	2.9
	October	0.14	0	0	0
2014 trial	July	0	0	0	0
	August	1.1	0	0	0
	September	9.9^∗^	21	9.8	9.8
	October	0	0	0	0
Correlation coefficient			0.597	0.555	0.576
*P*-value			0.04	0.06	0.05

*^∗^Considered as September value because the sampling time was behind plant rhizosphere sampling in August 2014.*

No *Salmonella* was isolated from the tomato fruits harvested in this experiment.

### *Salmonella* Recovered From Tomato Fields Under Different Fertilization and Production Practices (Experiment 2)

**FIGURE 4:**
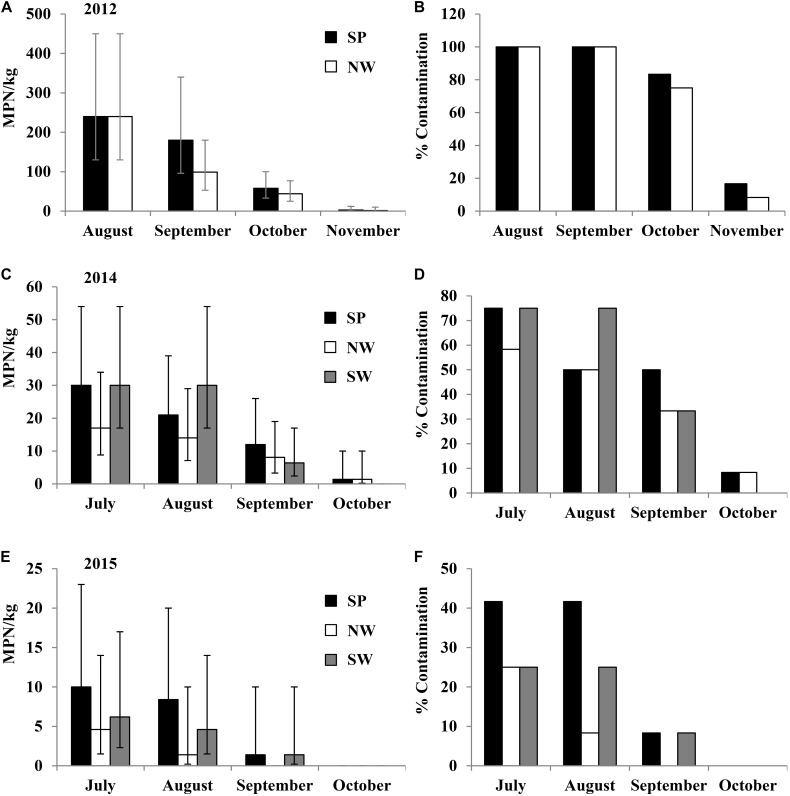
*Salmonella* population density (in 2012 **(A)**, 2014 **(C)**, and 2015 **(E)** field trials) and percent contaminated (in 2012 **(B)**, 2014 **(D)**, and 2015 **(F)** field trials) plant rhizosphere samples from tomato plots fertilized with fresh PL under different production practices in experiment 2. Bars represent 95% confidence intervals.

In the 2012 field trial, *Salmonella* population density and contamination ratio in plant rhizospheres of tomato plots, where PL was applied, decreased over time during the growth season from August to November. The initial average *Salmonella* population density in plant rhizospheres after soil amendment (with PL) was 240 MPN/kg (95% CI: 130–450 MPN/kg) under both production practices (**Figures 4A,B**). All the 12 plant rhizosphere samples tested in August and September in both SP and NW plots were *Salmonella* positive. Similar to experiment 1, *Salmonella* was isolated from the leaves of tomato plants in NW plots (leaves were in contact with soil) in September and October with 25 and 33% contaminated, respectively (**Supplementary Table S3**). *Salmonella* serovars Newport, Typhimurium, and Thompson were isolated from plant rhizosphere samples in the plots amended with PL, and Newport and Thompson were isolated from tomato leaf samples.

In the 2013 field trial, *Salmonella* was not isolated from the tomato fields (rhizosphere), leaf or fruit samples, even in the plots where PL was applied.

In the 2014 field trial, *Salmonella* was isolated from tomato plant rhizosphere that was treated with naturally contaminated PL. The initial average *Salmonella* population densities in plant rhizosphere samples were 30 (95% CI: 17–54 MPN/kg), 17 (95% CI: 8.8–34 MPN/kg), and 30 (95% CI: 17–54 MPN/kg) MPN/kg in July under production practices of SP, NW, and SW, respectively (**Figures 4C,D**). The initial contamination ratios were 75, 58.3, and 75% in SP, NW, and SW plots, respectively. Similar to the 2012 trial, *Salmonella* population density and contamination ratio in plant rhizosphere reduced during the growing season. Six of the 12 tomato leaf samples tested in NW plots were *Salmonella* positive in August (50%), and nine of the 12 NW leaf samples were positive in September (75%) (**Supplementary Table S3**). The top three *Salmonella* serovars isolated from plant rhizosphere samples in the plots amended with fresh PL were Kentucky (55%), Typhimurium (35%), and Newport (7%; *n* = 58). Only Kentucky and Typhimurium were isolated from tomato leaf samples in 2014 field trial.

In the 2015 field trial, *Salmonella* was only isolated from tomato plant rhizosphere samples in plots amended with fresh PL, with initial population density of 4.6–10 MPN/kg with 25–41.7% contaminated in July (**Figures 4E,F**). Similar to the observation in earlier trials, *Salmonella* population density and contamination ratio in plant rhizosphere decreased during the growing season. All tomato leaf samples tested in this trial were *Salmonella* negative. Typhimurium and Kentucky were the only two *Salmonella* serovars isolated from plant rhizosphere samples in the plots amended with PL in 2015 field trial.

In the four field trials of experiment 2, *Salmonella* was not isolated from plots fertilized with poultry liter ash or TSP. No contaminated fruits were detected in this experiment.

Based on the results derived from the six field trials of experiments 1 and 2, *Salmonella* population density and contamination ratio in plant rhizosphere samples were significantly higher in PL amended plots than that in pond water irrigated plots (*p* < 0.01).

### Contamination of *Salmonella* in Tomato Fields Using Different Irrigation Sources and Fertilizers (Experiment 3)

**FIGURE 5:**
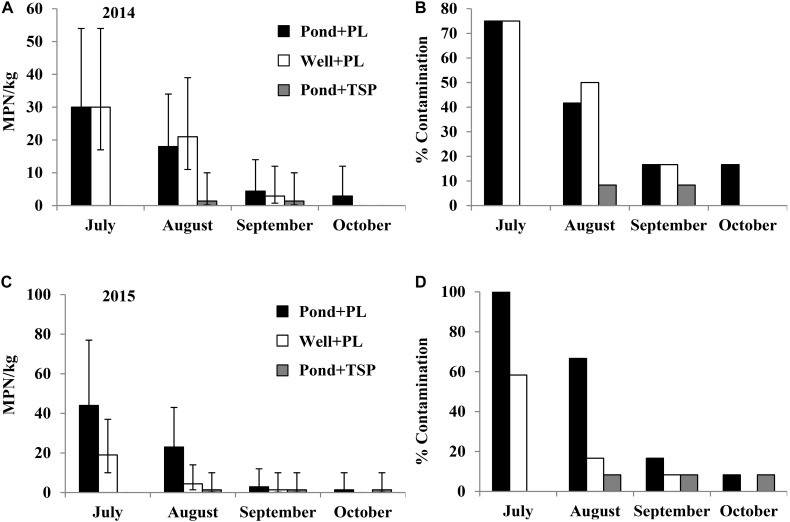
*Salmonella* population density (in 2014 **(A)**, and 2015 **(C)** filed trials) and percent contaminated (in 2014 **(B)**, and 2015 **(D)** filed trials) plant rhizosphere samples from experimental plots irrigated by Pond/Well water and fertilized with PL or triple superphosphate (TSP, conventional fertilizer) in experiment 3. Bars represent 95% confidence intervals.

In the 2014 field trial, *Salmonella* was isolated from nine of the 12 Pond+PL and Well+PL plant rhizosphere samples (75%) in July, with an average population density of 30 (95% CI: 17–54 MPN/kg) MPN/kg (**Figure 5A**). *Salmonella* population density and contamination ratio decreased under these two treatments during the growing season (**Figures 5A,B**). One of the 12 rhizosphere samples from Pond+TSP plots was *Salmonella* positive in August and September with a population density of 1.4 (95% CI: 0.2–10 MPN/kg) MPN/kg. Three of the 12 tomato leaf samples (25%) from Pond+PL plots were *Salmonella* positive in September (**Supplementary Table S4**). The major *Salmonella* serovars isolated from the plant rhizosphere samples were Kentucky (36%) and Saintpaul (26%) in Pond+PL plots (*n* = 58, **Supplementary Figure S3A**), and Kentucky (68%) and Typhimurium (26%) in Well+PL plots (*n* = 31, **Supplementary Figure S3B**). Newport and Saintpaul were the only two serovars isolated from plant rhizosphere samples in Pond+TSP plots, as well as contaminated leaf samples in Pond+PL plots.

In the 2015 field trial, *Salmonella* was only isolated from plant rhizosphere samples in Pond+PL, Well+PL and Pond+TSP plots. All the 12 Pond+PL samples (100%) and seven of the 12 Well+PL samples (58.3%) tested *Salmonella* positive in July, with population densities of 44 (95% CI: 25–77 MPN/kg) MPN/kg and 19 (95% CI: 10–37 MPN/kg) MPN/kg, respectively (**Figure 5C**). Similarly, *Salmonella* contamination ratio and population density decreased under these two treatments during the growth season (**Figure 5D**). One of the 12 rhizosphere samples from Pond+TSP plots was detected *Salmonella* positive from August to October with a population density of 1.4 (95% CI: 0.2–10 MPN/kg) MPN/kg. Different from the 2014 field trial, no tomato leaf samples were detected *Salmonella* positive in 2015. *Salmonella* serovars Kentucky and Typhimurium were isolated from plots fertilized with fresh PL (Pond+PL and Well+PL). Typhimurium and Newport were isolated from positive plant rhizosphere samples from Pond+TSP plots.

In the two field trials of experiment 3, no *Salmonella* was isolated from the plots irrigated by well water and applied with conventional fertilizer (Well+TSP). *Salmonella* was not isolated from the harvested tomato fruits in this experiment.

Average population density and contamination ratio of *Salmonella* in plant rhizosphere samples of the two trials were considerably higher in Pond+PL plots than that in Pond+TSP plots (*P* < 0.01), while the difference between Pond+PL and Well+PL samples was not significant (*P* = 0.34). *Salmonella* levels in Well+PL plots was relatively higher than that in Pond+TSP plots (*P* = 0.09). *Salmonella* population density and contamination ratio in plant rhizosphere samples of all the above three treatments (Pond+PL, Pond+TSP, and Well+PL) were significantly higher than that in Well+TSP plots, where no *Salmonella* was detected (*P* < 0.05).

### Contamination of *Salmonella* in Tomato Fields After Artificial Inoculation Using an Attenuated Strain (Experiment 4)

**FIGURE 6:**
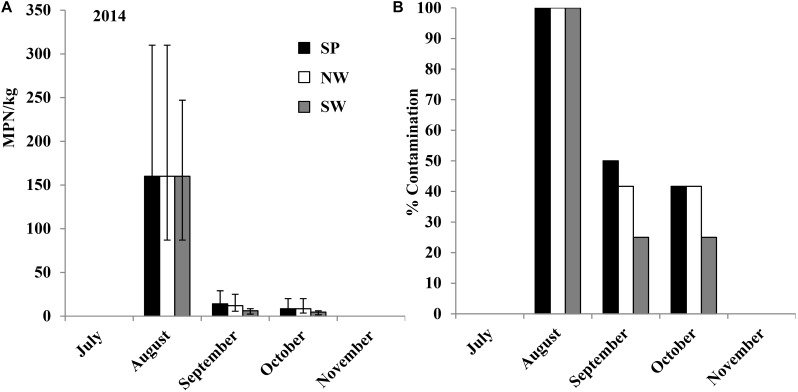
*Salmonella* population density **(A)** and percent contaminated **(B)** plant rhizosphere samples from tomato plots inoculated with attenuated *Salmonella* Newport strain SN#17 in the middle of August. The field trial of experiment 4 was performed in the growth season of 2014. Bars represent 95% confidence intervals.

After artificial inoculation in the 2014 field trail, plant rhizosphere soils in all inoculated plots were contaminated by the attenuated *Salmonella* Newport strain SN#17 with an initial average population density of 160 (95% CI: 87–310 MPN/kg) MPN/kg (0.16 MPN/g, 95% CI: 0.087–0.31 MPN/g) and showed a declining trend during the growing season (**Figures 6A,B**). Similar to the observation in experiments 1 and 2 (**Figures 3**, **4**), *Salmonella* MPN values and contamination ratios in plant rhizosphere were relatively higher in the plots covered with plastic mulch (SP). For tomato leaf samples, *Salmonella* SN#17 was isolated from six of the 12 samples collected from NW plots (leaves may touch soil as not staked) in August, September, and October (50%). Four tomatoes harvested from two of the four NW sub-plots (tomatoes not staked and no plastic mulch was used, tomatoes were observed on field soil) tested *Salmonella* positive. However, *Salmonella* was not detected from the fruits harvested from the inoculated SP and SW plots. The average contamination ratio on/in fruits was less than 1% (4 of 600 tested tomatoes). No *Salmonella* was isolated from control plots irrigated by well water without inoculation. Serotyping results confirmed that all *Salmonella* isolates from this experiment belong to the inoculated strain, serovar Newport.

## Discussion

Previous field studies revealed the effects of tomato cultivar, maturity, and various agronomical practices on the contamination of *Salmonella* on tomatoes, while in most of these researches, *Salmonella* infection was performed in the laboratory post-harvest (Xia et al., 2012; Han and Micallef, 2014; Marvasi et al., 2014, 2015; Devleesschauwer et al., 2017). In this study, irrigation pond water at ESAREC and fresh PL collected from local broiler farms were both found to be naturally contaminated with *Salmonella*. This served as a unique opportunity to investigate *Salmonella* survival and transmission in tomato fields by using naturally contaminated sources (**Table 1** and **Supplementary Table S1**).

Many previous studies have identified manure and irrigation water as potential contamination sources of *Salmonella* in fields (Natvig et al., 2002; Solomon et al., 2002; Islam et al., 2004a,b; Ohtomo et al., 2004; You et al., 2006; Bidol et al., 2007; Franz et al., 2008a,b; Semenov et al., 2009; Hintz et al., 2010; Park et al., 2012). In field trials using naturally contaminated sources (Experiments 1–3), soil amendments with fresh PL resulted in higher *Salmonella* population levels and contamination ratio in the plant rhizosphere, compared to irrigation with naturally contaminated pond water. There were statistically significant annual differences of *Salmonella* contamination ratio and population density in the three field experiments. The lower incidence of *Salmonella* in 2013 and 2014 field trials of experiment 1 (evaluation of irrigation and production practice) might be related to the total rainfall during the growth seasons, which may cause contamination through runoff water (**Supplementary Figure S4**). The total rainfall from July to October in 2013 (32.6 ± 2.4 cm) and 2014 (28.2 ± 2.1 cm) at ESAREC is lower than that during the field trial in 2012 (56.8 ± 12.5 cm). In experiments 2 (evaluation of fertilization and production practices) and 3 (evaluation of irrigation and fertilization), the initial population density of *Salmonella* in fresh PL amended into soils is the main factor that appeared to directly impact *Salmonella* MPN values at the beginning of each field trial in the plant rhizosphere in treated tomato plots and may also have affected the contamination ratio of leaf samples. The yearly shift of *Salmonella* strain diversity during the multi-year study may also contribute to the different prevalence of *Salmonella*, since the fitness of different *Salmonella* serotypes may vary.

A large increase in *Salmonella* spp. levels in pond water was observed in April (2013) and May (2014 and 2015). This increase might be associated with the increase in temperature, or potential applications of PL by neighboring farms for fertilization on non-edible crops (runoff into water source). Further studies to identify the original contamination source and to investigate the transmission and survival of foodborne pathogens in the ecosystem may inform prevention and mitigation strategies to limit *Salmonella* contamination on produce in this agricultural area.

In most field trials, *Salmonella* was isolated from tomato leaf samples collected only from plots that were not staked and plastic mulch was not used (NW plots); therefore, tomato plants were observed to readily contact soil. The consensus results in the multi-year study indicated that there is a higher risk for *Salmonella* transmission from contaminated soil to the above-ground part of tomato plants under practices that increase soil-plant contact (presumably because a larger part of plant leaves and fruits can directly contact the ground). In the 2014 trial of experiment 3, in which the combined effects of irrigation and fertilization were analyzed, *Salmonella* was isolated from a few tomato leaf samples in the plots covered with plastic mulch, which is different compared with the observation in other trails using naturally contaminated sources. The contamination route of *Salmonella* on tomato leaves and fruit is unclear. Rain splash and wild animals could be alternative reasons for the contamination (Gu et al., 2011; Cevallos-Cevallos et al., 2012; Jay-Russell, 2013; Gruszynski et al., 2014).

Artificial inoculation of attenuated *Salmonella* strain SN#17 did not result in significantly higher *Salmonella* contamination levels in plant rhizosphere in experiment 4 compared with several PL amended plots. This result may have been from the inability of the detection methods to accurately assess the concentration (MPN values) of *Salmonella* in plant rhizosphere and contamination ratio of rhizosphere and leaf samples due to the small inoculum amount applied to each plot. Only 2.5 L of the bacterial solution containing the attenuated *Salmonella* strain was inoculated once through drip tape to each sub-plot, containing about 30 tomato plants.

In this study, no *Salmonella* was isolated from tested PL ash samples or samples from plots fertilized with PL ash. PL ash has 3–6 times more phosphorus and potassium than fresh PL, which can be utilized as an alternative to other organic fertilizers (Codling et al., 2002; Reiter and Middleton, 2016). Conversion of fresh PL to ash through the heating process may provide a ‘kill step’ allowing vegetable growers to utilize PL as a fertilizer. More studies are needed to verify the efficacy of the PL ash production process.

Interestingly, *Salmonella* Newport, Typhimurium and Kentucky were isolated from the plant rhizosphere in the 2013 trial of experiment 1 (evaluation of irrigation and production practice). These serovars were not detected in sampled pond water during the same growth season. The differences in *Salmonella* serotypes among rhizosphere and pond water samples may arise from limited sample size and sampling frequency or indicate an additional source of contamination in the environment. The identified isolates in the study may not fully describe the whole serovar diversity in pond water and plant rhizosphere samples. In the 2013 field trial of experiment 2 (evaluation of fertilization and production practices), collected fresh PL was not immediately amended with breeding bed soils of sub-plots but stored in the collection bins for 2 days before application in the field. Composting might have occurred during the storing period which could have resulted in a significant reduction of *Salmonella* population in the fresh PL leading to negative detection results in the whole field trial. Some *Salmonella* isolates selected for molecular serotyping could not be identified or were grouped to two serotypes, such as Newport and Bardo, or uncommon categories, like Augustenborg and Kingston, especially in fresh poultry and plant rhizosphere samples. Additional phenotypic and molecular methods, like antibody microarrays, PFGE and whole genome sequencing, may facilitate further analysis of collected isolates.

The composition and population of *Salmonella* in naturally contaminated irrigation pond water and fresh PL shifted during the 4-year study. Newport was the most prevalent serovar of *Salmonella* isolated in pond water, while Typhimurium was the top one serovar that was isolated from fresh PL samples over the four consecutive years. The serovar composition in the two contamination sources were in agreement with former surveys conducted in this region (Micallef et al., 2012; Bell et al., 2015; Pagadala et al., 2015). The discrepancy between the *Salmonella* serovars isolated from different types of agricultural sources might be associated with the complex phenotypical and genetic properties that make the bacteria able to colonize and persist in the specific environment (Andino and Hanning, 2015). Future research to understand the survival abilities of certain *Salmonella* serovars in unique reservoirs may provide clues to improve current mitigation strategies.

The phenotypical and genetic properties of different *Salmonella* strains may also contribute to the transmission and survival of the pathogen in fields. The serovar diversity varied among different annual trials in the same field experiments applied with naturally contaminated sources. Further studies using whole genome sequencing analysis and antimicrobial susceptibility tests may help characterize *Salmonella* isolates from the various samples collected in this project. More sensitive subtyping should provide a deeper understanding of transmission and survival mechanisms of different *Salmonella* strains in agricultural environments. Estimation of *Salmonella* levels by MPN requires large sample volumes and numbers of replicates due to the low population density and uneven distribution of *Salmonella* in agricultural environments (Micallef et al., 2012; Pagadala et al., 2015); Another limitation of this study is the culture-based method may not be able to detect viable but non-culturable *Salmonella* cells, which may result in underestimation of bacterial population (Lee et al., 2015).

During the 4-year study, none of the 4,800 tomato fruits harvested from plots receiving naturally contaminated irrigation water or soil amendments were positive for *Salmonella*. When an attenuated *Salmonella* strain was added (at significantly higher levels of *Salmonella* than what was found in the naturally contaminated sources) to the soil through drip irrigation, a few cases of contaminated tomato fruits were detected (but only in plots without staking or plastic mulch) where fruits were in contact with the ground, with an average prevalence of 0.67%. Tomato production without staking and mulch is not routinely practiced by ESV growers and should be discouraged.

## Conclusion

This research provided science-based data to better understand pathogen contamination and transmission probability, and diversity dynamics of *S. enterica* serovars in fields using naturally contaminated pond irrigation water and fresh PL. Results derived from this study demonstrate that irrigation water and PL can serve as potential sources of foodborne pathogens for produce in agricultural fields. Data suggest that SP plots may limit the transmission of *Salmonella* from plant rhizosphere to above-ground parts. Application of contaminated PL may result in higher population densities of *Salmonella* in amended soils. Findings suggest tomatoes may become contaminated when in contact with the soil when soil was contaminated with *Salmonella*. Additionally, *Salmonella* serovar composition can vary among different environmental sources and samples, and the diversity can shift year to year. Potential future studies could probe the extent to which the phenotypic and genetic properties of different *Salmonella* strains may contribute to the colonization and survival of *Salmonella* in fields.

## Author Contributions

GG, DO, and SR studied the conception and design. GG, JZ, ER, and MR acquired the data. GG, DO, and LS analyzed and interpreted the data. GG drafted the manuscript. LS, DO, JZ, AO, RB, YC, SD, DI, RP, EB, and SR did the critical revision.

## Conflict of Interest Statement

The authors declare that the research was conducted in the absence of any commercial or financial relationships that could be construed as a potential conflict of interest.

## Supplementary Material

FIGURE S1 Diversity of *Salmonella enterica* serovars isolated from sampled pond water in 2013 **(A)** *n* = 20, 2014, **(B)** *n* = 16, 2015, **(C)** *n* = 32, and in total **(D)** *n* = 68. *n* denotes the number of identified isolates for each test.

FIGURE S2 Diversity of *S. enterica* serovars isolated from sampled fresh PL samples in 2012 **(A)** *n* = 70, 2014 **(B)** *n* = 39 and 2015 **(C)** *n* = 49. Typhimurium was the only *Salmonella* serovar identified in 2013 sampling (*n* = 40).

FIGURE S3 Diversity of *S. enterica* serovars isolated from plant rhizosphere samples in Pond+PL **(A)** *n* = 58 and Well+PL **(B)** *n* = 31 plots in 2014 field trial of experiment 3.

FIGURE S4 Dynamic changes in total rainfall and average temperature at the Virginia Tech ESAREC. **(A)** Monthly weather information from August 2012 to December 2013; **(B)** Weekly weather information in 2014; and **(C)** Weekly weather information in 2015.

TABLE S1 Sampling amount of tomato rhizosphere, leaf, and fruit samples for *Salmonella* detection in the field trials.

TABLE S2 *Salmonella* contamination of tomato leaves in plots irrigated by pond water in Experiment 1.

TABLE S3 *Salmonella* contamination of tomato leaves in plots fertilized with fresh PL in Experiment 2.

TABLE S4 *Salmonella* contamination of tomato leaves in Experiment 3.

## References

[B1] Allard S. Enurah A. Strain E. Millner P. Rideout S. L. Brown E. W. (2014). In situ evaluation of *Paenibacillus alvei* in reducing carriage of *Salmonella enterica* serovar Newport on whole tomato plants. *Appl. Environ. Microbiol.* 80 3842–3849. 10.1128/AEM.00835-14 24747888 PMC4054204

[B2] Andino A. Hanning I. (2015). *Salmonella enterica*: survival, colonization, and virulence differences among serovars. *Sci. World J.* 2015:520179. 10.1155/2015/520179 25664339 PMC4310208

[B3] Barak J. D. Kramer L. C. Hao L. Y. (2011). Colonization of tomato plants by *Salmonella enterica* is cultivar dependent, and type 1 trichomes are preferred colonization sites. *Appl. Environ. Microbiol.* 77 498–504. 10.1128/AEM.01661-10 21075871 PMC3020540

[B4] Bell R. L. Zheng J. Burrows E. Allard S. Wang C. Y. Keys C. E. (2015). Ecological prevalence, genetic diversity, and epidemiological aspects of *Salmonella* isolated from tomato agricultural regions of the Virginia Eastern Shore. *Front. Microbiol.* 6:415. 10.3389/fmicb.2015.00415 25999938 PMC4423467

[B5] Bennett S. D. Littrell K. W. Hill T. A. Mahovic M. Behravesh C. B. (2015). Multistate foodborne disease outbreaks associated with fresh tomatoes, United States, 1990–2010: a recurring public health problem. *Epidemiol. Infect.* 143 1352–1359. 10.1017/S0950268814002167 25167220 PMC9507195

[B6] Bidol S. A. Daly E. R. Rickert R. E. Hill T. A. Al Khaldi S. Taylor T. H. Jr. (2007). Multistate outbreaks of *Salmonella* infections associated with fresh tomatoes eaten in restaurants—United States, 2005–2006. *Morb. Mortal. Wkly. Rep.* 56 909–911. 17805221

[B7] Brandl M. T. (2006). Fitness of human enteric pathogens on plants and implications for food safety. *Annu. Rev. Phytopathol.* 44 367–392. 10.1146/annurev.phyto.44.070505.143359 16704355

[B8] Centers for Disease Control and Prevention [CDC] (2007). Multistate outbreaks of *Salmonella* infections associated with fresh tomatoes eaten in restaurants — United States, 2005-2006. *Morb. Mortal. Wkly. Rep.* 56 909–911. 17805221

[B9] Centers for Disease Control and Prevention [CDC] (2009). *Standard Protocol Molecular Determination of Serotype in Salmonella, Workshop on Molecular Determination of Serotype of Salmonella.* Atlanta, GA: Centers for Disease Control and Prevention.

[B10] Centers for Disease Control and Prevention [CDC] (2005). Outbreaks of *Salmonella* infections associated with eating Roma tomatoes—United States and Canada, 2004. *Can. Commun. Dis. Rep.* 31 225–228. 16669127

[B11] Centers for Disease Control and Prevention [CDC] (2006). *Salmonellosis—Outbreak Investigation.* Atlanta, GA: CDC.

[B12] Centers for Disease Control and Prevention [CDC] (2008). *Norovirus, Salmonella key Culprits in CDC Outbreak Report.* Atlanta, GA: CDC.

[B13] Cevallos-Cevallos J. M. Danyluk M. D. Gu G. Vallad G. E. van Bruggen A. H. C. (2012). Dispersal of *Salmonella* Typhimurium by rain splash onto tomato plants. *J. Food Prot.* 75 472–479. 10.4315/0362-028X.JFP-11-399 22410220

[B14] Clements M. (2016). *Burning Poultry Litter Creates Clean Energy for Producer. Wattagnet.com.* Available at: http://www.wattagnet.com/articles/25689-burning-poultry-litter-creates-clean-energy-for-producer [accessed April 28, 2017]

[B15] Codling E. E. Chaney R. L. Sherwell J. (2002). Poultry litter ash as a potential phosphorous source for agricultural crops. *J. Environ. Qual.* 31 954–961. 10.2134/jeq2002.9540 12026100

[B16] Danyluk M. (2013). Produce and irrigation water quality: are EPA standards appropriate for fresh produce application? *IAFP Annu. Meet. Abstr.* 102 S1–S2.

[B17] Devleesschauwer B. Marvasi M. Giurcanu M. C. Hochmuth G. J. Speybroeck N. Havelaar A. H. (2017). High relative humidity pre-harvest reduces post-harvest proliferation of *Salmonella* in tomatoes. *Food Microbiol.* 66 55–63. 10.1016/j.fm.2017.04.003 28576373

[B18] Food and Drug Administration [FDA] (2017). *Archive for Recalls, Market Withdrawals & Safety Alerts.* Available at: http://www.fda.gov/Safety/Recalls/default.htm [accessed May 10, 2017]

[B19] Franz E. Semenov A. V. Termorshuizen A. J. de Vos O. J. Bokhorst J. G. van Bruggen A. H. C. (2008a). Manure-amended soil characteristics affecting the survival of *E-coli* O157:H7 in 36 Dutch soils. *Environ. Microbiol.* 10 313–327. 10.1111/j.1462-2920.2007.01453.x 18199123

[B20] Franz E. Semenov A. V. van Bruggen A. H. C. (2008b). Modelling the contamination of lettuce with *Escherichia coli* O157:H7 from manure-amended soil and the effect of intervention strategies. *J. Appl. Microbiol.* 105 1569–1584. 10.1111/j.1365-2672.2008.03915.x 19146493

[B21] Franz E. van Bruggen A. H. (2008c). Ecology of *E. coli* O157:H7 and *Salmonella enterica* in the primary vegetable production chain. *Crit. Rev. Microbiol.* 34 143–161. 10.1080/10408410802357432 18728991

[B22] Greene S. K. Daly E. R. Talbot E. A. Demma L. J. Holzbauer S. Patel N. J. (2008). Recurrent multistate outbreak of *Salmonella* Newport associated with tomatoes from contaminated fields, 2005. *Epidemiol. Infect.* 136 157–165. 10.1017/S095026880700859X 17475091 PMC2870807

[B23] Gruszynski K. Pao S. Kim C. Toney D. Wright K. Ross P. G. (2014). Evaluating wildlife as a potential source of *Salmonella* serotype Newport (JJPX01.0061) contamination for tomatoes on the eastern shore of Virginia. *Zoonoses Public Health* 61 202–207. 10.1111/zph.12061 23773825

[B24] Gu G. Cevallos-Cevallos J. M. van Bruggen A. H. C. (2013). Ingress of *Salmonella enterica* into tomato leaves through hydathodes. *PLoS One* 8:e53470. 10.1371/journal.pone.0053470 23320087 PMC3540056

[B25] Gu G. Hu J. Cevallos- Cevallos J. M. Richardson S. M. Bartz J. A. van Bruggen A. H. C. (2011). Internal colonization of *Salmonella enterica* serovar Typhimurium in tomato plants. *PLoS One* 6:e27340. 10.1371/journal.pone.0027340 22096553 PMC3212569

[B26] Gu G. Zheng J. Reiter M. Strawn L. Rideout S. L. (2015). Prevalence and survival of Salmonella enterica spp. in irrigation water, poultry litter and amended soils on the Eastern Shore of Virginia. *Int. Assoc. Food Protect. Annu. Meet. Abstr* 104 T7–T12.

[B27] Habetz D. Echols R. (2006). Development of successful poultry litter-to-energy furnace. *Paper Presented Written for 2006 ASABE Annual International Meeting*, Detroit, MI.

[B28] Han S. Micallef S. A. (2014). *Salmonella* Newport and Typhimurium colonization of fruit differs from leaves in various tomato cultivars. *J. Food Prot.* 77 1844–1850. 10.4315/0362-028X.JFP-13-562 25364916

[B29] Hanning I. B. Nutt J. D. Ricke S. C. (2009). Salmonellosis outbreaks in the United States due to fresh produce: sources and potential intervention measures. *Foodborne Pathog. Dis.* 6 635–648. 10.1089/fpd.2008.0232 19580447

[B30] Hintz L. D. Boyer R. R. Ponder M. A. Williams R. C. Rideout S. R. (2010). Recovery of *Salmonella enterica* Newport introduced through irrigation water from tomato (*Lycopersicum esculentum*) fruit, roots, stems, and leaves. *HortScience* 45 675–678.

[B31] Islam M. Morgan J. Doyle M. P. Phatak S. C. Millner P. Jiang X. (2004a). Fate of *Salmonella enterica* serovar Typhimurium on carrots and radishes grown in fields treated with contaminated manure composts or irrigation water. *Appl. Environ. Microbiol.* 70 2497–2502. 10.1128/AEM.70.4.2497-2502.2004 15066849 PMC383101

[B32] Islam M. Morgan J. Doyle M. P. Phatak S. C. Millner P. Jiang X. (2004b). Persistence of *Salmonella enterica* serovar Typhimurium on lettuce and parsley and in soils on which they were grown in fields treated with contaminated manure composts or irrigation water. *Foodborne Pathog. Dis.* 1 27–35. 10.1089/153531404772914437 15992259

[B33] Jay-Russell M. T. (2013). *What is the Risk from wild Animals in Food-borne Pathogen Contamination of Plants?* Wallingford: CABI.

[B34] Lee K. M. Runyon M. Herrman T. J. Phillips R. Hsieh J. (2015). Review of *Salmonella* detection and identification methods: aspects of rapid emergency response and food safety. *Food Control* 47 264–276. 10.1016/j.foodcont.2014.07.011

[B35] Luo Z. Gu G. Ginn A. van Bruggen A. H. C. Danyluk M. Wright A. (2016). Distribution and characterization of *Salmonella enterica* isolates from irrigation ponds in southeastern U.S.A. *Appl. Environ. Microbiol.* 81 8243–8253. 10.1128/AEM.04086-14 25911476 PMC4475880

[B36] Luo Z. Gu G. Giurcanu M. C. Adams M. Vellidis G. van Bruggen A. H. C. (2014). Development of a novel cross-streaking method for isolation, confirmation, and enumeration of *Salmonella* from irrigation ponds. *J. Microbiol. Methods* 101 86–92. 10.1016/j.mimet.2014.03.012 24732066

[B37] Maguire R. O. Heckendorn S. E. (2015). *Soil Test Recommendations for Virginia.* Available at: http://www.soiltest.vt.edu/PDF/recommendation-guidebook.pdf [accessed April 8, 2016]

[B38] Marvasi M. George A. S. Giurcanu M. C. Hochmuth G. J. Noel J. T. Gause E. (2014). Effects of nitrogen and potassium fertilization on the susceptibility of tomatoes to post-harvest proliferation of *Salmonella enterica*. *Food Microbiol.* 43 20–27. 10.1016/j.fm.2014.03.017 24929878

[B39] Marvasi M. George A. S. Giurcanu M. C. Hochmuth G. J. Noel J. T. Teplitski M. (2015). Effect of the irrigation regime on the susceptibility of pepper and tomato to post-harvest proliferation of *Salmonella enterica*. *Food Microbiol.* 46 139–144. 10.1016/j.fm.2014.07.014 25475277

[B40] Micallef S. A. Rosenberg Goldstein R. E. George A. Kleinfelter L. Boyer M. S. McLaughlin C. R. (2012). Occurrence and antibiotic resistance of multiple *Salmonella* serotypes recovered from water, sediment and soil on mid-Atlantic tomato farms. *Environ. Res.* 114 31–39. 10.1016/j.envres.2012.02.005 22406288

[B41] Miles J. M. Sumner S. S. Boyer R. R. Williams R. C. Latimer J. G. McKinney J. M. (2009). Internalization of *Salmonella enterica* serovar Montevideo into greenhouse tomato plants through contaminated irrigation water or seed stock. *J. Food Prot.* 72 849–852. 10.4315/0362-028X-72.4.849 19435236

[B42] Natvig E. E. Ingham S. C. Ingham B. H. Cooperband L. R. Roper T. R. (2002). *Salmonella enterica* serovar Typhimurium and *Escherichia coli* contamination of root and leaf vegetables grown in soils with incorporated bovine manure. *Appl. Environ. Microbiol.* 68 2737–2744. 10.1128/AEM.68.6.2737-2744.2002 12039728 PMC123957

[B43] Ohtomo R. Minato K. Saito M. (2004). Survival of *Escherichia coli* in a field amended with cow feces slurry. *Soil Sci. Plant Nutr.* 50 575–581. 10.1080/00380768.2004.10408514

[B44] Opara O. O. Carr L. E. Russek-Cohen E. Tate C. R. Mallinson E. T. Miller R. G. (1992). Correlation of water activity and other environmental conditions with repeated detection of *Salmonella* contamination on poultry farms. *Avian Dis.* 36 664–671. 10.2307/1591762 1417596

[B45] Pagadala S. Marine S. C. Micallef S. A. Wang F. Pahl D. M. Melendez M. V. (2015). Assessment of region, farming system, irrigation source and sampling time as food safety risk factors for tomatoes. *Int. J. Food Microbiol.* 196 98–108. 10.1016/j.ijfoodmicro.2014.12.005 25540859

[B46] Painter J. A. Hoekstra R. M. Ayers T. Tauxe R. V. Braden C. R. Angulo F. J. (2013). Attribution of foodborne illnesses, hospitalizations, and deaths to food commodities by using outbreak data, United States, 1998-2008. *Emerg. Infect. Dis.* 19 407–415. 10.3201/eid1903.111866 23622497 PMC3647642

[B47] Park S. Szonyi B. Gautam R. Nightingale K. Anciso J. Ivanek R. (2012). Risk factors for microbial contamination in fruits and vegetables at the preharvest level: a systematic review. *J. Food Prot.* 75 2055–2081. 10.4315/0362-028X.JFP-12-160 23127717

[B48] Pope M. J. Cherry T. E. (2000). An evaluation of the presence of pathogens on broilers raised on poultry litter treatment-treated litter. *Poult. Sci.* 79 1351–1355. 10.1093/ps/79.9.1351 11020084

[B49] Reiter M. Middleton A. (2016). “Farm manure-to-energy initiative in the chesapeake region report January 2016,” in *Appendix F: Nutrient Availability from Poultry Litter Co-Products*, (Painter, VA: Virginia Tech Eastern Shore Agricultural Research and Extension Center), 120–190.

[B50] Semenov A. V. van Overbeek L. van Bruggen A. H. C. (2009). Percolation and survival of *Escherichia coli* O157:H7 and *Salmonella enterica* serovar Typhimurium in soil amended with contaminated dairy manure or slurry. *Appl. Environ. Microbiol.* 75 3206–3215. 10.1128/AEM.01791-08 19270130 PMC2681632

[B51] Solomon E. B. Yaron S. Matthews K. R. (2002). Transmission of *Escherichia coli* O157:H7 from contaminated manure and irrigation water to lettuce plant tissue and its subsequent internalization. *Appl. Environ. Microbiol.* 68 397–400. 10.1128/AEM.68.1.397-400.2002 11772650 PMC126537

[B52] Statista (2015). *Top 10 Fresh Market Tomato Producing U.S. States in 2013.* Available at: http://www.statista.com/statistics/193242/top-10-fresh-market-tomato-producing-states-in-the-us/ [accessed September 12, 2016]

[B53] Strawn L. K. Gröhn Y. T. Warchocki S. Worobo R. W. Bihn E. A. Wiedmann M. (2013). Risk factors associated with *Salmonella* and *Listeria monocytogenes* contamination of produce fields. *Appl. Environ. Microbiol.* 13 3982–3991. 10.1128/AEM.02831-13 24077713 PMC3837806

[B54] Suresh T. Hathab A. A. M. Harshac H. T. Lakshmanaperumalsamya P. (2011). Prevalence and distribution of *Salmonella* serotypes in marketed broiler chickens and processing environment in Coimbatore City of southern India. *Food Res. Int.* 44 823–825. 10.1016/j.foodres.2011.01.035

[B55] United States Department of Agriculture [USDA] (2009). *Vegetables and Melons: Tomatoes [online]. USDA, Economic Research Service.* Available at: https://www.ers.usda.gov/topics/crops/vegetables-pulses/tomatoes.aspx#Fresh tomato [accessed May 17, 2017]

[B56] van Asselt E. D. Thissen J. T. N. M. van der Fels-Klerx H. J. (2009). *Salmonella* serotype distribution in the Dutch broiler supply chain. *Poult. Sci.* 88 2695–2701. 10.3382/ps.2009-00074 19903970

[B57] Volkova V. V. Bailey R. H. Wills R. W. (2009). *Salmonella* in broiler litter and properties of soil at farm location. *PLoS One* 4:e6403. 10.1371/journal.pone.0006403 19636431 PMC2712689

[B58] Wilson S. P. Kuhar T. P. Rideout S. L. Freeman J. H. Reiter M. S. Straw R. A. (2012). *Commercial Vegetable Production Recommendations.* Blacksburg, VA: Virginia Cooperative Extension.

[B59] Xia X. Luo Y. Yang Y. Vinyard B. Schneider K. Meng J. (2012). Effects of tomato variety, temperature differential, and post-stem removal time on internalization of *Salmonella enterica* serovar Thompson in tomatoes. *J. Food Prot.* 75 297–303. 10.4315/0362-028X.JFP-11-078 22289590

[B60] You Y. Rankin S. C. Aceto H. W. Benson C. E. Toth J. D. Dou Z. (2006). Survival of *Salmonella enterica* serovar newport in manure and manure-amended soils. *Appl. Environ. Microbiol.* 72 5777–5783. 10.1128/AEM.00791-06 16957193 PMC1563654

[B61] Zheng J. Allard S. Reynolds S. Millner P. Arce G. Blodgett R. J. (2013). Colonization and internalization of *Salmonella enterica* in tomato plants. *Appl. Environ. Microbiol.* 79 2494–2502. 10.1128/AEM.03704-12 23377940 PMC3623171

