# Supplementary figures and images for: Agricultural Practices Influence Salmonella Contamination and Survival in Pre-harvest Tomato Production
Source: Front Microbiol. 2018 Oct 16;9:2451. doi: 10.3389/fmicb.2018.02451 (PMC6198144; doi:10.3389/fmicb.2018.02451)

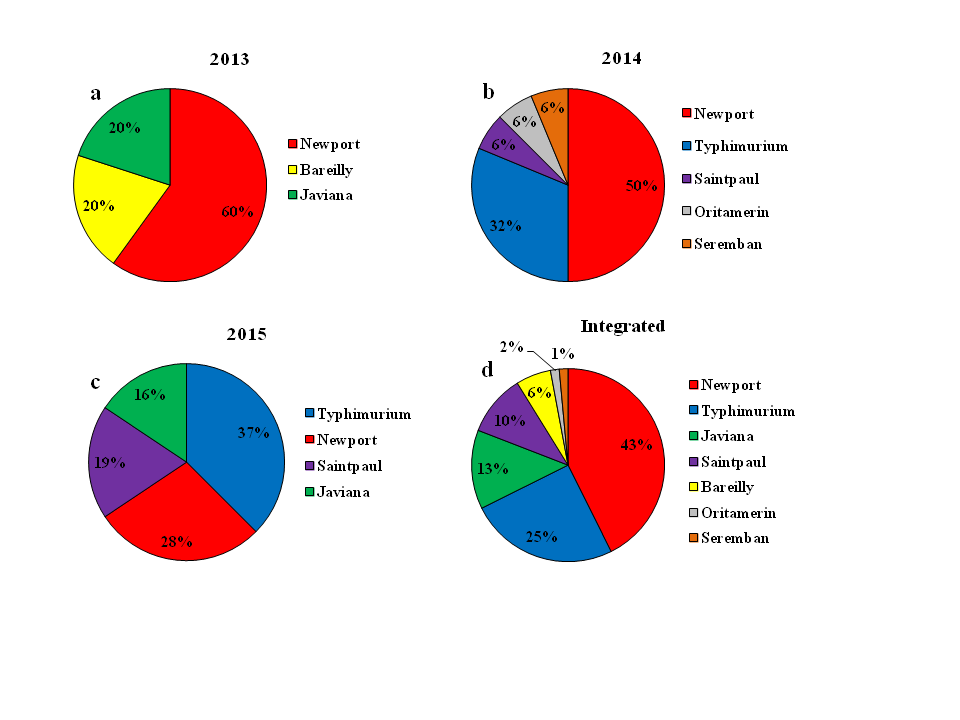

Supplement: FIGURE S1 — Diversity of Salmonella enterica serovars isolated from sampled pond water in 2013 (A) n = 20, 2014, (B) n = 16, 2015, (C) n = 32, and in total (D) n = 68. n denotes the number of identified isolates for each test. [file Image_1.TIF]

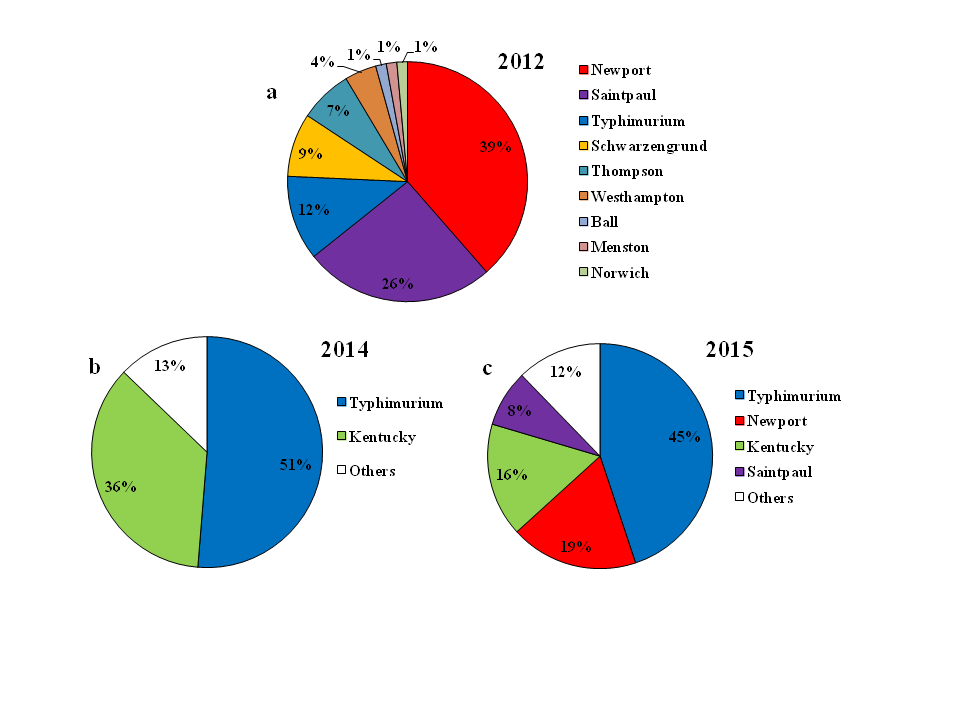

Supplement: FIGURE S2 — Diversity of S. enterica serovars isolated from sampled fresh PL samples in 2012 (A) n = 70, 2014 (B) n = 39 and 2015 (C) n = 49. Typhimurium was the only Salmonella serovar identified in 2013 sampling (n = 40). [file Image_2.TIF]

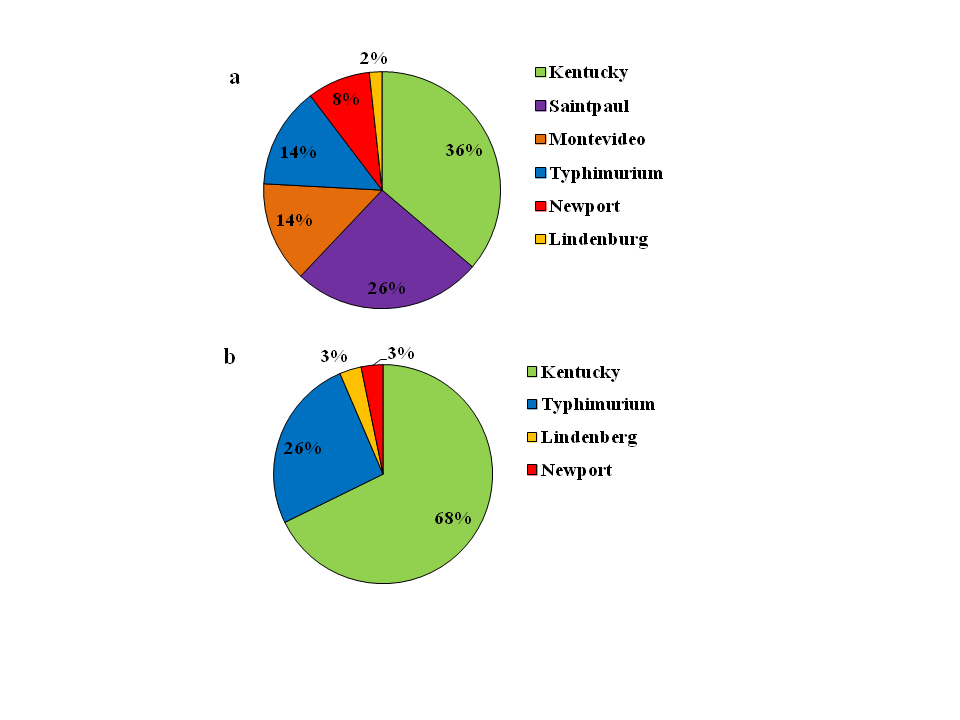

Supplement: FIGURE S3 — Diversity of S. enterica serovars isolated from plant rhizosphere samples in Pond+PL (A) n = 58 and Well+PL (B) n = 31 plots in 2014 field trial of experiment 3. [file Image_3.TIF]

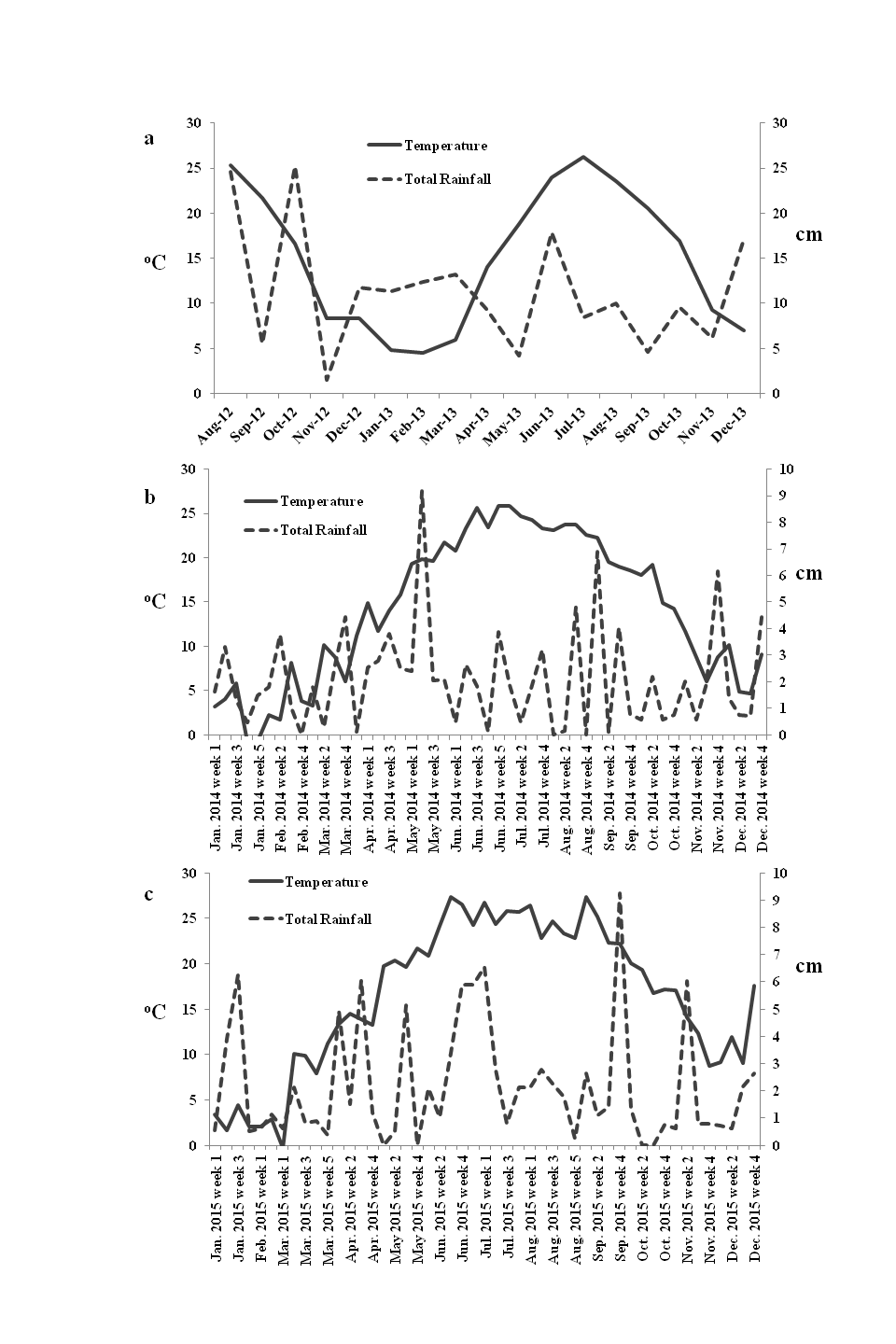

Supplement: FIGURE S4 — Dynamic changes in total rainfall and average temperature at the Virginia Tech ESAREC. (A) Monthly weather information from August 2012 to December 2013; (B) Weekly weather information in 2014; and (C) Weekly weather information in 2015. [file Image_4.TIF]
