# Supplementary material for: Agricultural Practices Influence Salmonella Contamination and Survival in Pre-harvest Tomato Production
Source: Front Microbiol. 2018 Oct 16;9:2451. doi: 10.3389/fmicb.2018.02451 (PMC6198144; doi:10.3389/fmicb.2018.02451)
Supplement: TABLE S1 — Sampling amount of tomato rhizosphere, leaf, and fruit samples for Salmonella detection in the field trials. [file Data_Sheet_1.docx]

**Table S1.** Sampling size of tomato rhizosphere, leaf, and fruit samples for *Salmonella* detection in tomato field experiments.

| Experiment | Trial | Tested rhizosphere* and leaf samples | Tested Fruits |
| --- | --- | --- | --- |
| 1 | 2012 | 3 composite samples/subplot × 4 replications × 6 treatments × 4 months = 288 samples | 25 fruits/subplot × 4 replications × 2 sampling times × 6 treatments = 1200 fruits |
|  | 2013 | 3 composite samples/subplot × 4 replications × 6 treatments × 4 months = 288 samples | 25 fruits/subplot × 4 replications × 2 sampling times × 6 treatments = 1200 fruits |
|  | 2014 | 3 composite samples/subplot × 4 replications × 6 treatments × 4 months = 288 samples | 25 fruits/subplot × 4 replications × 2 sampling times × 6 treatments = 1200 fruits |
| 2 | 2012 | 3 composite samples/subplot × 4 replications × 6 treatments × 4 months = 288 samples | 25 fruits/subplot × 4 replications × 2 sampling times × 6 treatments = 1200 fruits |
|  | 2014 | 3 composite samples/subplot × 4 replications × 9 treatments × 4 months = 432 samples | 25 fruits/subplot × 4 replications × 2 sampling times × 9 treatments = 1800 fruits |
|  | 2015 | 3 composite samples/subplot × 4 replications × 9 treatments × 4 months = 432 samples | 25 fruits/subplot × 4 replications × 2 sampling times × 9 treatments = 1800 fruits |
| 3 | 2014 | 3 composite samples/subplot × 4 replications × 4 treatments × 4 months = 192 samples | 25 fruits/subplot × 4 replications × 2 sampling times × 4 treatments = 800 fruits |
|  | 2015 | 3 composite samples/subplot × 4 replications × 4 treatments × 4 months = 192 samples | 25 fruits/subplot × 4 replications × 2 sampling times × 4 treatments = 800 fruits |
| 4 | 2014 | 3 composite samples/subplot × 4 replications × 6 treatments × 5 months = 360 samples | 25 fruits/subplot × 4 replications × 2 sampling times × 6 treatments = 1200 fruits |

*, a MPN method was used to quantify *Salmonella* population in tomato rhizosphere samples.

**Table S2.** *Salmonella* contamination of tomato leaves in plots irrigated by pond water in Experiment 1.

|  | Number of leaves testing positive for *Salmonella* | | | | |
| --- | --- | --- | --- | --- | --- |
|  | July | August | September | October | November |
| 2012 trial | NA | 0 | 1* | 0 | 0 |
| 2013 trial | 0 | 0 | 0 | 0 | NA |
| 2014 trial | 0 | 0 | 4 | 0 | NA |

*, 12 tomato leaf samples were tested per month during each field trial. NA, not available.

**Table S3.** *Salmonella* contamination of tomato leaves in plots fertilized with fresh PL in Experiment 2.

|  | Number of leaves testing positive for *Salmonella* | | | | |
| --- | --- | --- | --- | --- | --- |
|  | July | August | September | October | November |
| 2012 trial | NA | 0 | 3* | 4 | 0 |
| 2013 trial | 0 | 0 | 0 | 0 | NA |
| 2014 trial | 0 | 6 | 9 | 0 | NA |
| 2015 trial | 0 | 0 | 0 | 0 | NA |

*, 12 tomato leaf samples were tested per month during each field trial. NA, not available.

**Table S4.** *Salmonella* contamination of tomato leaves in Experiment 3.

|  | Number of leaves testing positive for *Salmonella* | | | | |
| --- | --- | --- | --- | --- | --- |
|  |  | August | September | October | November |
| 2014 trial | Pond+PL | 0 | 3* | 0 | 0 |
|  | Pond+TSP ^a^ | 0 | 0 | 0 | 0 |
|  | Well+TSP | 0 | 0 | 0 | 0 |
|  | Well+PL | 0 | 0 | 0 | 0 |
| 2015 trial | Pond+PL | 0 | 0 | 0 | 0 |
|  | Pond+TSP | 0 | 0 | 0 | 0 |
|  | Well+TSP | 0 | 0 | 0 | 0 |
|  | Well+PL | 0 | 0 | 0 | 0 |

*, 12 tomato leaf samples were tested per month during each field trial. ^a^, Conventional fertilizer triple superphosphate.
